# Identification and Characterization of HD1, a Novel Ofloxacin-Degrading *Bacillus* Strain

**DOI:** 10.3389/fmicb.2022.828922

**Published:** 2022-03-03

**Authors:** Jing Zhang, Naiqing Sha, Yanhong Li, Shen Tang, Yuqing Peng, Yao Zhao

**Affiliations:** College of Environmental Science and Engineering, Guilin University of Technology, Guilin, China

**Keywords:** ofloxacin, separation and screening, metabolomics, degradation pathway, *Bacillus* sp.

## Abstract

In recent years, an increasing number of lakes and soils around the world have been polluted by antibiotics, seriously threatening the ecological balance and human health. Currently, there is a lack of understanding of the biodegradation mechanism of typical antibiotics by microorganisms. In this study HD1, a novel *Bacillus* sp. strain called capable of effectively degrading ofloxacin (OFL), a typical antibiotic with a high detection rate in the environment, was isolated from soil contaminated by OFL. The results of single-factor experiments showed that the optimal conditions for OFL degradation included 30°C, pH 7.0, and 10 g L^–1^ NaCl. After 7 days of incubation under aerobic conditions, the degradation efficiency of OFL (5 mg L^–1^) was about 66.2%. Five degradation products were detected by LC-MS analysis, and it was deduced that the possible degradation pathways of OFL included the oxidation of the piperazine ring, demethylation, hydroxylation, and methoxy cleavage. Metabolomics analysis indicated that key pathways with the highest difference with HD1 metabolites included the phenylalanine, arginine, and proline metabolism pathways. By regulating energy, amino acid metabolism, and carbohydrate metabolism, HD1 could alleviate OFL stress to degrade better. This study explored the degradation mechanism of OFL by HD1 and provides a theoretical basis and technical support for the remediation of OFL-contaminated environments by functional microorganisms.

## Introduction

Ofloxacin (OFL), a third-generation fluoroquinolone (FQ) antibiotic, is internationally recognized as an extremely important broad-spectrum antimicrobial agent that is widely used for the clinical treatment of infections and diseases caused by Gram-negative bacteria (Aggio et al., 2010). In recent decades, aqueous environments around the world have increasingly been polluted by FQs (Karthikeyan and Meyer, 2006; Yang et al., 2018). In 11 rivers in Hong Kong, China, OFL was one of the main antibiotics detected (Deng et al., 2018). As a semi-synthetic antibiotic, the stable chemical properties of OFL make it resistant to biodegradation even after decades (Schulz et al., 2019). In addition, due to its high solid-water partition coefficient, it is also easily enriched on the surface of various stable environmental particle carriers, including atmospheric dust, soil particles, and aqueous sediments (Golet et al., 2003; Zhao et al., 2016; Reis et al., 2020). Accumulation and retention of antibiotics in the environment can promote the selection of resistance genes in bacterial populations and affect the dynamics of biological populations followed by entering the human body through the biological chain, which may lead to high risks for ecosystems and human health (Xu et al., 2015; Yang et al., 2018). Therefore, the economical and effective remediation of antibiotic pollution of the environment has become a research hotspot (Maki et al., 2006). In fact, compared with physical and chemical remediation methods, antibiotics in most environments are more likely to be decomposed or transformed by microorganisms (Schulz et al., 2019). Microbial remediation of OFL in the environment is one of the most economical and effective methods (Liu et al., 2016). Screening contaminated environments for bacterial strains that are resistant-specific and that are able to efficiently degrade antibiotics is one of the most important means of remediation. The efficient microbial degradation of antibiotics is dependent on environmental factors such as pH, temperature, salt content, and redox conditions (Arikan et al., 2009; Liu et al., 2016). Therefore, the rate of biodegradation of OFL can be improved by screening for strains that efficiently degrade OFL followed by optimizing the environmental conditions of degradation.

Microbial metabolomics studies the complete metabolic characteristics of a biological system by examining the composition and content changes of all small-molecule metabolites before and after a stimulation or disturbance of the biological system in a specific period. It can also be used to identify biomarkers and metabolic pathways (Geng et al., 2016). Due to its high precision, high resolution, high sensitivity, and high throughput, it has been widely used for the detection of microbial metabolites, metabolic pathway research, and microbial classification and identification (Zhang, 2017). Liu et al. (2021) studied the effects of microplastics and dichlorodiphenyltrichloroethane (DDT) on *E. coli* through metabolomics. The results showed that the toxic effect of DDT was significantly dose-dependent, while the presence of microplastics weakened the response of DDT to the growth and metabolism of *E. coli*. The tricarboxylic acid cycle-related enzyme activity and antioxidant defense-related substances in *E. coli* also confirmed this mechanism. Li et al. (2020) revealed that biochar combined with plant roots improved bacterial stress to polycyclic aromatic hydrocarbons by metabolomics. Metabolomics has allowed the field of environmental research to make great progress in the past few years and it is considered to be a powerful tool for environmental safety assessment (Zhao et al., 2011). In addition, through the identification of degradation products, it is of great significance to clarify the degradation pathway of pollutants in microorganisms and for elucidating the mechanism of microbial remediation. Durand et al. (2010) used *in situ* nuclear magnetic resonance and liquid chromatography- nuclear magnetic resonance as supplementary tools for LC-MS to determine the metabolic pathway of mesotrione (a new herbicide) degradation by *Bacillus* sp.

Although progress has been made in the understanding of the environmental fate of antibiotics, little is known about the metabolic potential and degradation mechanism of specific antibiotics by environmental functional microorganisms. The purpose of this study was (1) to screen and isolate high-efficiency OFL degrading bacterial strains from OFL-contaminated soil and identify them by 16S rRNA. (2) To explore the optimal conditions for OFL degradation and its possible degradation pathways by HPLC-Q-TOF-MS analysis. (3) The use of metabolomics-based methods to find the differences in metabolites, and then explore the changes in metabolic pathways under OFL stress to determine the degradation mechanism.

## Materials and Methods

### Main Reagents

The mixed soil samples were collected from the surrounding area of a livestock and poultry farm in Guilin, Guangxi. All chemical reagents used in the experiments were of analytical grade. Ultrapure water was used throughout. Main media: (1) Inorganic salt medium: K_2_HPO_4_ 5.8 g L^–1^, KH_2_PO_4_ 4.5 g L^–1^, (NH_4_)_2_SO_4_ 2.0 g L^–1^, MgCl_2_ 0.16 g L^–1^, and CaCl_2_ 0.02 g L^–1^, adjusted to pH 7.0. (2) Basic medium: tryptone 10.0 g L^–1^, yeast extract 5.0 g L^–1^, NaCl 10.0 g L^–1^, pH 7.5. The medium was sterilized at 121°C for 30 min before use. (3) Phosphate buffer: NaCl 8 g L^–1^, KCl 0.2 g L^–1^, KH_2_PO_4_ 0.2 g L^–1^, pH 7.0. (4) OFL standard stock solution: 1.0 g OFL was added into 100 mL deionized water and stirred in the dark with a magnetic stirrer until completely dissolved. The OFL stock solution was stored shielded from light at 4°C.

### Domestication of Bacteria and Isolation and Purification of Strains

The domestication of OFL-degrading bacteria referred to existing methods (Reis et al., 2014) with slight adjustments. The process was divided into three stages and each stage had a domestication cycle of 15 days. The first stage was as follows: under sterile conditions, soil samples (3 g) were mixed with 100 mL inorganic salt medium with yeast extract (1.0 g L^–1^) as the carbon source. The bacteria were cultured in a constant temperature oscillation incubator at 30°C and 150 rpm for 15 days in the dark. Add quantitative OFL to make the initial concentration of 5 mg/Land concentration of OFL was gradually increased to 30 mg L^–1^ with increments of 5 mg L^–1^. At the same time, the concentration of yeast extract was decreased gradually from 1.0 g L^–1^, to 0.5, 0.3, 0.2, and finally to 0.1 g L^–1^. In each enrichment step, 100 ml of freshly prepared sterilized inorganic salt medium was inoculated with 1% of the volume of the culture in the previous stage. Enriched bacteria (ECI) were obtained after domestication. In the second stage, the ECI bacteria were inoculated (1%) into inorganic salt medium and the carbon source in the medium was replaced by a mixture containing amino acids, vitamins, and adenine. The domestication method was the same as in the first stage, and the enriched bacteria obtained were designated ECII. The procedure was repeated in the third stage with an amino acid mixture as carbon source, with the other conditions remaining unchanged, to obtain ECIII. The residual concentrations of OFL in the media at different domestication stages were determined at regular intervals by HPLC and the degradation rates were calculated to screen for bacteria that degrade OFL with high efficiency (Wang, 2020) (see the Supplementary Material for details).

Following acclimation, the activated high-efficiency OFL-degrading bacteria were collected by centrifugation at 2,504 *g* for 5 min. The cells were washed 2–3 times with phosphate buffered saline (PBS) and then resuspended in PBS. Dilutions (10^–4^ ∼ 10^–8^) of the bacterial suspension were evenly smeared on the agar solid medium containing OFL. The cells were cultured at 30°C for 3 ∼ 6 days until the colonies grew out. Single colonies with different morphological characteristics were selected and further purified by the streak plate method. The procedure was repeated 3 ∼ 4 times until five pure strains (named HD1, HD2, HD3, HD4, and HD5, respectively) were obtained. These were inoculated into inorganic salt medium containing OFL (1 mg L^–1^), and their OFL degradation activity was tested after 7 days. The experiment of inorganic salt medium containing OFL without bacteria was designed as the control to eliminate the interference of OFL spontaneous degradation.

### Morphological Observation of Strains and Homology Analysis of 16S rDNA Sequences

The morphology of the strains was characterized by scanning electron microscopy (SEM). The cells cultured for more than 6 h were removed from the 24-well plate, washed three times with PBS, and fixed with 2.5% glutaraldehyde for 24 h. Gradient dehydration with ethanol was carried out at subsequent concentrations of 30, 50, 70, 80, 90, and 100% ethanol for 10 min each, followed by freeze-drying for 8 h. The cell mounting pieces were fixed to the sample table with conductive adhesive for gold spraying and the morphology of the cells was observed by SEM (Lu et al., 2018).

Genomic DNA was extracted using the OMEGA genomic DNA extraction kit. Next, 16S rDNA fragments were PCR-amplified with universal primers 27F (AgAgTTTgATCCTg CTCAg) and 1492R (TACgg(C/T)TACCTTgTTACgACTT). PCR reaction mix (30 μL): 2 × PCR Mix 15 μL, DNA 1 μL, upstream primer (10 mM) 1 μL, downstream primer (10 mM) 1 μL, ddH_2_O 12 μL. Reaction conditions: 95°C 5 min, 94°C 45 s, 55°C 45 s, 72°C 1 min 15 s, 32 cycles, 72°C 10 min. After the reaction was completed, the size and specificity of the amplified fragments were verified by 1% agarose gel electrophoresis and photographed using a gel imaging system. The PCR amplification products were purified and sequenced by Beijing Orwellson Biotechnology Co., Ltd. The 16S rDNA sequencing results of the obtained strains were analyzed using BLAST, and the related sequences with high homology were selected for relationship analysis. A phylogenetic tree was constructed by the neighbor method of MEGA5 software.

### Activity of Ofloxacin-Degrading Bacteria

Strain HD1 was inoculated (1%) into inorganic salt medium (pH 7.0) containing OFL (5 mg L^–1^). The effects of temperature (15, 25, 30, 35, and 45°C), pH (4, 5, 6, 7, 8, 9, and 10) and NaCl concentration (0, 5, 10, 15, and 20 mg L^–1^) on the degradation of OFL by HD1 were investigated. Three groups of controls were set in the experiment.

### Sample Preparation and Mass Spectrometry Analysis

The supernatant of the inorganic salt medium was filtered using a 0.22 μm filter membrane, and the filtrate was analyzed by high-resolution mass spectrometry (HPLC-Q-TOF-MS). The molecular weight of the intermediate product was determined, and the material structure was assessed by analyzing the molecular weight of the intermediate product in the mass spectrum.

Degradation products were analyzed using LC-MS or LC-Q-TOF-MS. The high-resolution mass spectrometer was equipped with an electrospray ion source (ESI) ion source and a quadrupole flight device. The analysis was carried out in the negative ion mode with the scanning mode of 1/2 MS 100 ∼ 1,000. The sample injection volume was 20 μL, and the flow rate was 0.3 ml min^–1^. The chromatographic column was Agilent Extend-C18 (2.1 mm × 50 mm, 1.7 μm). The mobile phase was 0.1% formic acid solution (mobile phase A) and acetonitrile (mobile phase B). Gradient elution was as follows: 10% mobile phase B: 1 min. The mobile phase B increased to 90% within 3 min and this was maintained for 12 min. Next, the mobile phase B decreased to 10% within 3 min. The ion source gas temperature was 350°C, the ion gas concentration was 12 mL/min, the ion spray voltage was 4,000 V, the first-order mass spectrometry collision energy is 175 V, and the second-order mass spectrometry collision energy is 185 V.

The degradation products of OFL were analyzed by HPLC-Q-TOF-MS. Under positive ion mode, after 7 days of culture, the bacterial liquid was injected into the mass spectrometry by injection pump at the speed of 0.3 ml min^–1^. The response intensity of ESI to OFL molecular ions was investigated in positive and negative ion mode by full scan mode, and the parent ion was determined.

### Sampling and Extraction of Metabolites

A suspension of the HD1 strain (1 mL) was inoculated into 100 mL LB liquid medium and cultured at 35°C for 24 h at 150 r min^–1^. In the experimental group, the OFL stock solution was added into 100 mL LB medium, at final concentrations of OFL of 10 and 5 mg L^–1^ recorded as group A and group B, respectively, with five parallel samples in each group, denoted as A1 ∼ A5 and B1 ∼ B5. The control group lacked OFL, marked as C1 ∼ C5. After 24 h of culture, the appropriate amount of bacterial liquid was collected and placed in a sterile centrifuge tube followed by centrifugation at 2,504 *g*, 4°C for 3 min. The bacteria were resuspended in pre-cooled PBS, centrifuged for 3 min at 2,504 *g* and 4°C, and then frozen on dry ice.

Extraction of metabolites was conducted according to Sangster et al. (2006) and Aggio et al. (2010). Microbial cell metabolomics by methyl chloroformate derivatization-gas chromatography-mass spectrometry was as follows: samples (60 mg) were transferred to a 2 mL centrifuge tube and 500 μL methanol (−20°C) were added as well as 500 μL Double distilled water (ddH_2_O) (4°C), and glass beads (100 mg), and vortexed for 30 s. The centrifuge tube was placed in an adapter, immersed in liquid nitrogen for 5 min, and then thawed at room temperature. Next, the centrifuge tube was replaced in the adapter, placed in a tissue grinder, and oscillated at 70 Hz for 2 min. This procedure was repeated twice. The sample was centrifuged at 7,512 *g* for 10 min at 4°C. The supernatant was concentrated, and dried. The sample was dissolved in 300 μL of a 2-chlorophenylalanine (4 ppm) methanol solution (1:1, 4°C) and filtered through a 0.22 μm membrane. The sample was then analyzed by LC-MS. Aliquots (20 μL) were used as quality control (QC) samples, and the remaining samples were analyzed by LC-MS.

Chromatographic conditions were as follows (Holmes et al., 2010): gas chromatography was performed on a Thermo Vanquish instrument, equipped with an ACQUITY UPLC^®^ HSS T3 1.8 μm (2.1 mm × 150 mm) chromatographic column. The temperature of the automatic sampler was set to 8°C, the flow rate was 0.25 ml min^–1^, the column temperature was 40°C, and the injection volume was 2 μL for gradient elution. The mobile phase was 0.1% formic acid aqueous solution (A1)—0.1% formic acid acetonitrile (B1). The negative ion was a 5 mmol L^–1^ ammonium formate aqueous solution (A2) and acetonitrile (B2). The gradient elution program was as follows: 0 ∼ 1 min, 2% B2/B1. 1 ∼ 9 min, 2 ∼ 50% B2/B1. 9 ∼ 12 min, 50 ∼ 98% B2/B1. 12 ∼ 13.5 min, 98% B2/B1. 13.5 ∼ 14 min, 98 ∼ 2% B2/B1. 14 ∼ 20 min, 2% B1—positive mode (4 ∼ 17 min, 2% B2—negative mode).

## Results and Discussion

### Isolation and Identification of Ofloxacin-Degrading Bacteria

**FIGURE 1:**
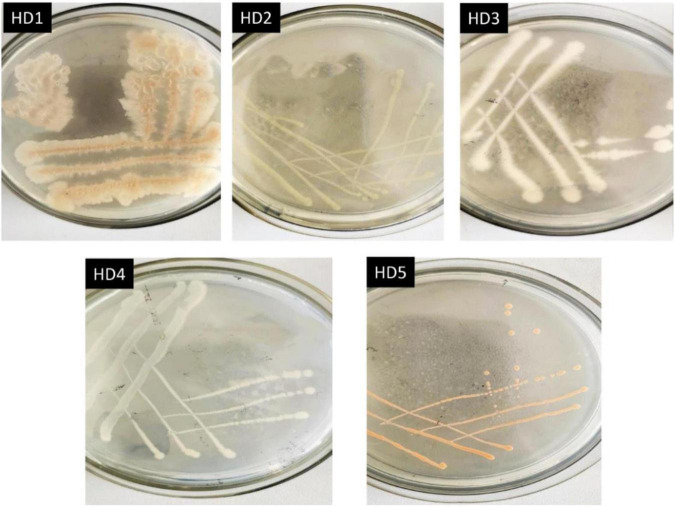
Colony morphologies of 5 OFL-resistant bacterial strains on solid medium.

The activated high-efficiency OFL-degrading bacterium after domestication was streaked on an agar plate, and single colonies with good growth and consistent morphology were purified by continuous streaking. Five strains of OFL-resistant bacteria were obtained after 3 ∼ 4 purification cycles, which were labeled HD1, HD2, HD3, HD4, and HD5, respectively. The colony morphologies of the five strains are shown in Figure 1 and the 16S rDNA gene sequences were obtained and compared with GeneBank sequences. The results of the comparison are shown in Supplementary Table 1.

**FIGURE 2:**
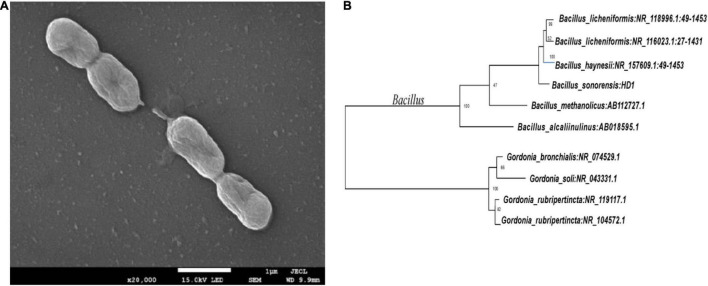
**(A)** Scanning Electron Microscope image of HD1 and **(B)** Phylogenetic tree based on 16S rRNA sequence highlighting the phylogenetic position of HD1.

The five OFL-degrading strains were inoculated into the inorganic salt medium (pH 7.0) containing OFL (5 mg L^–1^), grown at 30°C and tested for OFL degradation activity within 7 days. We found that the OFL degradation efficiency of HD1 was 66.2%, while that of the other 4 strains was 28.4, 61.9, 45.6, and 58.4%, respectively. And the degradation rate of the control group was 2.16% (Supplementary Figure 1). The SEM images showed that the cells of strain HD1 were rod-shaped, flagella-free, wrinkled, and convex in the middle, while the surface was rough and glossy. The dimensions of the cells were 1.2–1.3 μm × 0.8–1.0 μm (Figure 2A). The strain was identified as gram-positive and facultative anaerobic following the protocol described by experimental method of “Common Bacterial System Identification Manual” and “Bergey’s Manual of Determinative Bacteriology” with slight adjustments (Hort et al., 1994; Dong and Cai, 1999). The 16S rRNA amplicon sequencing results revealed a total of 1,404 bases with a homology between HD1 and *Bacillus haynesii* of 99.575%. The location of HD1 in a phylogenetic tree based on 16S rDNA sequences is shown in Figure 2B. Previous studies have found that *Bacillus* strains can degrade norfloxacin, a different fluoroquinolone antibiotic, effectively, mainly through the structures of the pyrazine ring and the quinolone ring (Liyanage and Manage, 2018).

### Effect of pH, Temperature, and NaCl Concentration on the Degradation Rate of Ofloxacin by Strain HD1

**FIGURE 3:**
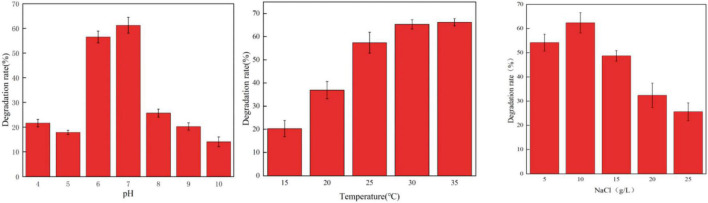
The Effect of different pH values (left, 30°C, 10 g/L NaCl), temperatures (middle, PH = 7, 10 g/L NaCl), and NaCl concentrations (right, 30°C, PH = 7) on the degradation rate of OFL by strain HD1.

Environmental conditions such as temperature and pH value can significantly affect the degradation rate of a variety of antibiotics such as chlortetracycline (CT), oxytetracycline (OTC), and tetracycline (TET) (Feng et al., 2013; Böttger et al., 2015). In this study, the effects of pH, temperature, and NaCl concentration on the degradation of OFL by HD1 were investigated under different environmental conditions (Figure 3). It was found that when pH was in the range of 6.0 ∼ 7.0, the strain could carry out normal growth and metabolism, and its ability to degrade OFL was also relatively stable. However, below pH 6.0 or above pH 7.0, the ability of HD1 to degrade OFL by HD1 had significantly worsened. When the temperature was 25 ∼ 35°C, the strain metabolized well, and the degradation effect of OFL was good at 30°C. When the temperature was ≤ 15°C, the degradation efficiency of OFL was the lowest, which may be due to the stagnation of growth and metabolism of strains at low temperatures. When the salt concentration range was 5 ∼ 15 g L^–1^, the growth and metabolism of the strain were normal, and the optimal salinity for degrading OFL was 10 g L^–1^. As studies have shown, high concentrations of salt in river water and activated sludge inhibit the biodegradation of sulfonamides (Li and Zhang, 2010; Adamek et al., 2016). Studies have also found that in pure fresh water (salinity of 0%) and pure sea water (salinity of 34.82%), the conversion rate of Vibrio vulgaris L2-2 to a variety of sulfonamide antibiotics is low (Wang, 2021). We infer that too high or too low salinity will affect the osmotic pressure of bacteria, resulting in slow growth and metabolism of strains, and the degradation of OFL will also decrease.

### Analysis of Degradation Products and Pathway Inference

Current studies have shown that the degradation mechanisms of FQs mainly include the following pathways: piperazine ring cracking, demethylation, hydroxylation, and N-acetylation. However, the specific biodegradation pathway of OFL remains unclear. Wang et al. (2012) used HPLC-MS to identify the degradation products of levofloxacin due to damage by acid, oxidation, and light. Four intermediate degradation products were obtained with mass-to-core ratios of 328.1, 378.1, 348.2, and 378, respectively. Golet et al. (2003) studied the degradation of norfloxacin by the norfloxacin-degrading strain *Staphylococcus caprae* and found four degradation products produced by the following pathways: breaking and oxidation of the piperazine ring, replacement of the fluorine atom by a hydroxyl group or benzene ring hydrogen. In this experiment, molecular ion peaks with high response intensity were observed in the mass spectrum (Supplementary Figure 2). According to the ion characteristic peak at m/z = 362.1519 [M + H]^+^ and its molecular weight of 361.1529, the substance was determined as OFL. The characteristic peaks of four ion fragments in the secondary mass spectrum were m/z = 261.1033, 318.1612, 314.1299, and 332.1401, respectively. We speculated that the molecular formulae of the main degradation products during the degradation of OFL by strain HD1 are C_17_H_21_FN_3_O_2_, C_14_H_14_FN_2_O_2_, C_17_H_21_FN_3_O_3_, C_17_H_19_FN_3_O_2_, and C_12_H_8_FN_2_O_2_, respectively, and the molecular structure of OFL is shown in Supplementary Table 2.

**FIGURE 4:**
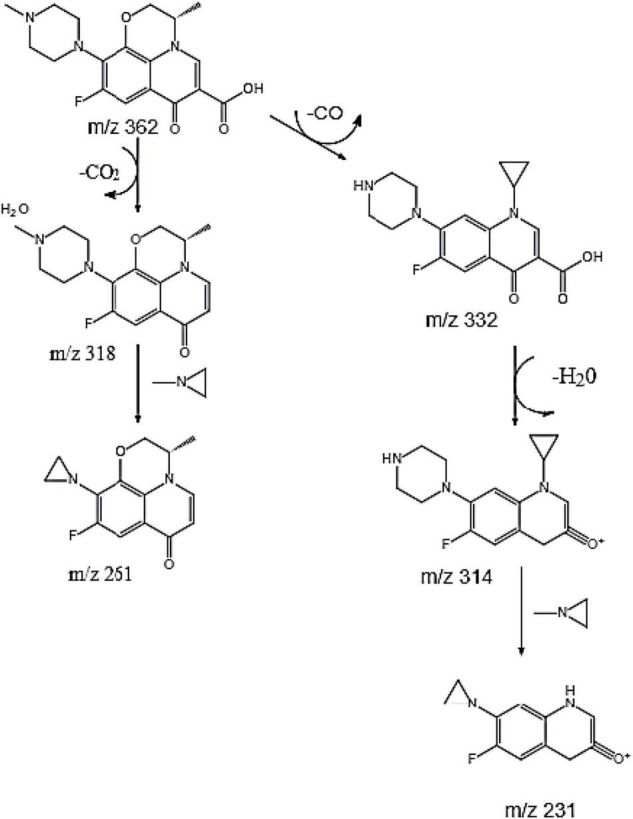
Possible degradation pathways of OFL.

Based on the above analysis, we speculated that there are two parallel pathways for the degradation of OFL by the HD1 strain (Figure 4). In the first possible pathway, the carbonyl group of OFL was removed by an inducible enzyme to form the product C_17_H_21_FN_3_O_2_. Then, the carbon-nitrogen bond on the piperazine ring was oxidized to form an 1-Methylethylenimine, and finally the degradation product C_14_H_14_FN_2_O_2_. In the second possible pathway OFL lost a C atom and C = O bond and the methyl group and the methoxy group of the benzene ring on the piperazine ring were broken. Following the ring-opening, C_17_H_21_FN_3_O_3_ was rearranged. Previous studies on the degradation of ciprofloxacin (CIP) found that CIP degradation mainly occurred on two functional groups: the piperazine group and quinolone part, and the product was also detected in the mass spectrometry ion fragments (Hongguang et al., 2016). Then an H_2_O molecule was removed from C_17_H_21_FN_3_O_3_ to form C_17_H_19_FN_3_O_2_. Finally, the C-N bond on the benzene ring was broken, the amino ring ethyl was removed, and the piperazine ring was oxidized to form C_12_H_8_FN_2_O_2_. This product was also detected in the degradation products of norfloxacin and CIP by a white-rot fungus (Prieto et al., 2011). Based on this, we inferred that the degradation pathways of OFL include piperazine ring oxidation, demethylation, hydroxylation, and methoxy breakage.

### Metabolomics Analysis of HD1 Under Ofloxacin Stress

**FIGURE 5:**
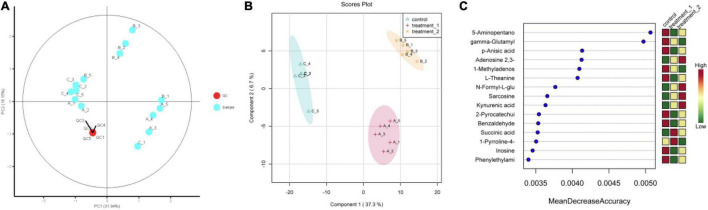
**(A)** Quality control sample PCA chart; **(B)** PLS-DA point cloud; **(C)** variable importance in project corresponding to the PLS-DA. The abscissa of **(C)** (Mean Decrease Accuracy) measures the importance of a metabolite in random forests. The right figure is the heat map of the content of metabolites in the two groups in 15.) Treatment 1 corresponds to group A (OFL concentration is 10 mg/L); treatment 2 corresponds to group B (OFL concentration is 5 mg/L); Control does not add OFL.

**FIGURE 6:**
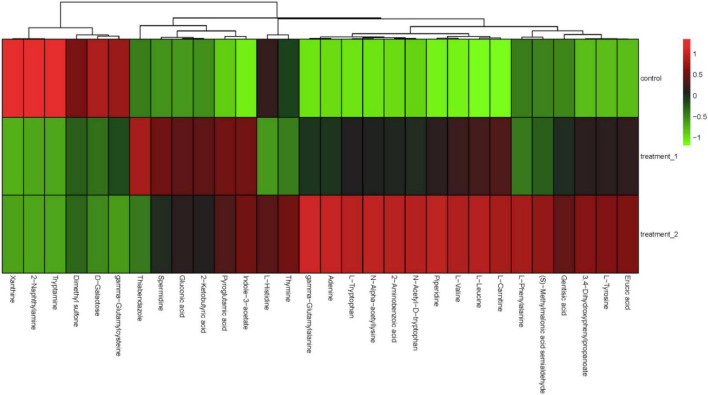
Hierarchical cluster analysis heat map. The concentration of 10 mg/L OFL is treatment 1; and the concentration of 5 mg/L OFL is treatment 2; Control does not add OFL.

The QC-RFSC algorithm of the Stat Target package of R language was used for QC to correct the signal peak of each sample and record the correction effect. As shown in Figure 5A, the QC sample points overlapped and all samples were located in a 95% confidence interval, which strongly suggested that the experimental data are highly reliable. Partial least squares discriminant analysis (PLS-DA) projected the predicted and observed variables into a new space, as shown in Figure 5B, indicating that different OFL concentrations led to significant changes in metabolite patterns. Therefore, we speculated that strain HD1 adapted to different OFL concentrations with different metabolic response mechanisms. Some evidences of bacterial metabolomics regulation indicate that the high abundance of bacterial endogenous metabolites is closely related to the resistance of bacteria to antibiotics. Such as indole, nitric oxide, hydrogen sulfide, glucose, and amino acids can change the metabolic environment of a variety of bacterial cells and antibiotic sensitivity (Gusarov et al., 2009; Allison et al., 2011; Shatalin et al., 2011). In this study, the strongest positively correlated metabolites according to random forest classification were 5-aminopentano, gamma-glutamyl, p-anisic acid, 1-methyladenosine, and L-theanine, while adenosine 2,3 was negatively correlated with OFL concentrations (Figure 5C). Therefore, the significant changes in these metabolites may also indicate that they may be a key biomarker for the resistance of strain HD-1 to OFL. These results prompted us to explore whether the suppressed level of adenosine 2,3 is a useful biomarker and if adenosine 2,3 could act as a modulator for OFL-resistant. The thermogram of metabolite changes showed that amino acid metabolites (such as L-phenylalanine and L-tryptophan) were responsible for the changes in metabolic patterns, which might be due to the significant up-regulation of these metabolites upon OFL treatment (Figure 6). Since L-tryptophan is often hidden inside the protein, its indole ring is very sensitive to the environment, so it can be used as an indicator of protein structural changes (Chen and Barkley, 1998; Zhang et al., 2015). Therefore, it can also be inferred that OFL treatment changed the protein structure of the strain.

**FIGURE 7:**
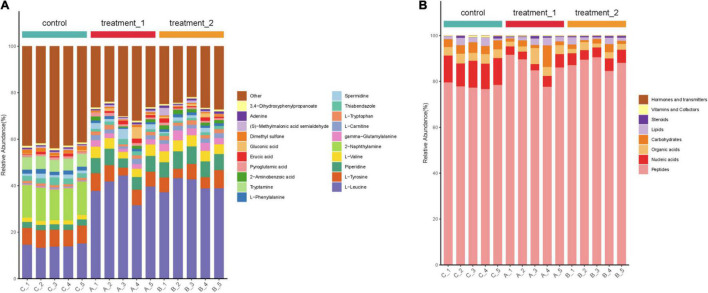
**(A)** Stacked column chart indicating relative abundance of metabolites or **(B)** groups of metabolites. Shown in the left panel is the percentage accumulation of the top 20 metabolites upon treatment with OFL at two different concentrations compared with the control treatment. The right panel shows the relative abundance of metabolites according to biological roles. The concentration of 10 mg/L OFL is treatment 1; and the concentration of 5 mg/L OFL is treatment 2; Control does not add OFL.

According to the percentage accumulation column diagram of metabolites (Figure 7) the metabolite content of strain HD1 was significantly different in OFL stress environments compared with the absence of OFL. Under OFL stress, the content of L-leucine and piperidine increased significantly while the content of Tryptamine, 2-naphthylamine decreased compared with the control. The significant changes in metabolite levels indicated that OFL stress may induce changes in multiple metabolic pathways in HD1. For example, the increased amino acid (L-leucine) content may be the result of increased catabolism generated by energy. Under external pressure, proteins are decomposed to release free amino acids or peptides (Fu et al., 2019). This was confirmed by Figure 7 (right panel), showing that the relative abundance of peptides metabolized by HD1 in the presence of OFL was higher than that of the control group. The decreased relative abundance of nucleic acids and carbohydrates may indicate that OFL stimulated glycolysis and nucleotide metabolism in HD1 and increased microbial metabolic activity accelerated intracellular carbohydrate decomposition and energy consumption to adapt to environmental disturbances (Hu et al., 2020). Microbial metabolism is a complex regulatory network involving multiple genes and proteins. Microorganisms can adjust their metabolic processes to cope with environmental pressure to improve their chances of survival (Kosová et al., 2018).

**FIGURE 8:**
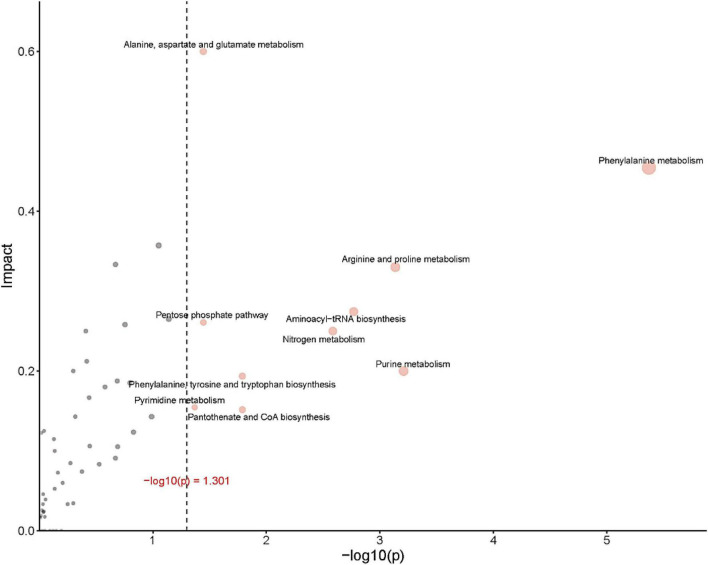
ORA (enrichment analysis) and topological analysis diagram. (Horizontal coordinates indicate the *p*-value for ORA analysis. The area indicates significant differences (*p* < 0.05). The ordinate indicates topological analysis impact).

Figure 8 provides an over-representation (enrichment) analysis (ORA) of the pathways that were affected by OFL treatment, indicating the pathway impact value [-log (p)] determined by metabolic pathway enrichment analysis and metabolic pathway topology analysis. The pathway analysis, using MetaboAnalyst, showed that the observed metabolic disturbance indicated the effects on various pathways, including phenylalanine metabolism and arginine and proline metabolism, alanine, aspartate and glutamate metabolism, pentose phosphate pathway, nitrogen metabolism, purine metabolism, and aminoacyl-tRNA biosynthesis, further indicating that the effects on these pathways may affect the energy metabolism, amino acid metabolism, and carbohydrate metabolism of HD1. Recent studies have reached similar conclusions. Researchers have found that the abundance of L-aspartic acid is related to the antibiotic resistance of A. hydrophila. L-aspartic acid is inhibited in the metabolism of strains, and this study shows that by regulating the metabolic pathway of L-aspartic acid, Change the sensitivity of bacteria to antibiotics (Zhao et al., 2018). In addition, the results further showed that phenylalanine, arginine, and proline metabolism pathways were the key pathways in HD1 that were most affected by OFL treatment (Figure 8).

## Conclusion

In this work, we isolated a novel *Bacillus* strain, named HD-1, that effectively degraded OFL from among high-efficiency degrading bacteria enriched in OFL-contaminated soil. Under optimal conditions (30°C, pH 7.0, 10 g L^–1^NaCl), the degradation rate of OFL was 66.2%. We further detected five OFL degradation products by HPLC-MS/MS and proposed two putative OFL degradation pathways in HD1. The main degradation processes involved the oxidation, demethylation, and hydroxylation of the piperazine ring of OFL. Through the analysis and identification of differential metabolites and metabolomics analysis of HD1 under OFL stress, we found that the differences between metabolite levels between normal (non-stressed) HD1 and OFL-stressed HD1 was highest in three key metabolic pathways, suggesting that HD1 could alleviate OFL stress by regulating energy, amino acid metabolism, and carbohydrate metabolism. The results of this study are critical for evaluating the fate of OFL in engineering and natural systems and for designing bioremediation strategies. In addition, this study expands the existing knowledge of microbial degradation in ecosystems affected by OFL pollution and provides a theoretical basis for the application of bioremediation in an environment with high antibiotic levels.

## Data Availability Statement

The original contributions presented in the study are included in the article/Supplementary Material, further inquiries can be directed to the corresponding author/s.

## Author Contributions

JZ and NS designed and conducted the experiments. YL and ST compiled and analyzed the output data, designed and wrote the first version of the manuscript, and conceived and supervised the project. YP and YZ managed the funding acquisition. All authors edited and approved the final version of the manuscript.

## Conflict of Interest

The authors declare that the research was conducted in the absence of any commercial or financial relationships that could be construed as a potential conflict of interest.

## Publisher’s Note

All claims expressed in this article are solely those of the authors and do not necessarily represent those of their affiliated organizations, or those of the publisher, the editors and the reviewers. Any product that may be evaluated in this article, or claim that may be made by its manufacturer, is not guaranteed or endorsed by the publisher.
