# Corrigendum: Identification and characterization of HD1, a novel ofloxacin-degrading *Bacillus* strain

**DOI:** 10.3389/fmicb.2022.952884

**Published:** 2022-08-09

**Authors:** Jing Zhang, Naiqing Sha, Yanhong Li, Shen Tang, Yuqing Peng, Yao Zhao

**Affiliations:** College of Environmental Science and Engineering, Guilin University of Technology, Guilin, China

**Keywords:** ofloxacin, separation and screening, metabolomics, degradation pathway, *Bacillus* sp.

In the published article there was an error in the **Funding** statement. The correct number for “the National Science Foundation of China” is “No. 52070050.”

The authors apologize for this error and state that this does not change the scientific conclusions of the article in any way. The original article has been updated.

## Publisher's note

All claims expressed in this article are solely those of the authors and do not necessarily represent those of their affiliated organizations, or those of the publisher, the editors and the reviewers. Any product that may be evaluated in this article, or claim that may be made by its manufacturer, is not guaranteed or endorsed by the publisher.

