# Supplementary material for: Identification and Characterization of HD1, a Novel Ofloxacin-Degrading Bacillus Strain
Source: Front Microbiol. 2022 Mar 3;13:828922. doi: 10.3389/fmicb.2022.828922 (PMC8928261; doi:10.3389/fmicb.2022.828922)
Supplement: Supplementary file 1 [file Data_Sheet_1.docx]

**Supplementary material**

**1. HPLC detection conditions**

In order to detect the remaining OFL concentration of the samples during the test, samples are taken regularly, pretreated with a solid phase extraction cartridge, and then quantitatively detected by HPLC. The specific operation method is as follows:

Accurately measure 15 mL of the culture solution, filter it with a 0.45 μm glass fiber membrane, adjust the pH to 3.0 with 4 mol·L^-1^ sulfuric acid, and then clean the HLB solid phase extraction column at a flow rate of 5 mL·min^-1^ (used 6 mL methanol and 6 mL ultrapure water activation). After the water sample passes through the chromatographic column, wash it with 6ml ultrapure water and 6ml 5 % methanol aqueous solution, and then dry the solid phase extraction column in vacuum for 30 min. Then elute with 6 ml of methanol (under natural gravity flow), blow it to near dryness with nitrogen, dilute it to 1 ml with methanol, and use 0.22 μM membrane filter to 2 ml Brown sample bottle and store at -20 ℃.

The test conditions of high performance liquid chromatography are: C18 column (4.6×150 mm, 5μm), column temperature 25 ℃; mobile phase A: acetonitrile, mobile phase B: 0.2% acetic acid aqueous solution, and A: B=15: 85, the flow rate is 1 mL·min^-1^, the injection volume is 20 μL; the UV detector wavelength is 288 nm.

**2. Preliminary Identification and** **Degradability determination of OFL Efficient Degrading Bacteria**

**Table S1. The homology comparison results of the gene sequences of 5 OFL-resistant bacteria in GeneBank**

| Number | | Homology comparison results | Belonging to the genus |
| --- | --- | --- | --- |
| HD1 | *Bacillus haynesii*（99.57%） | | *Bacillus* |
| HD2 | *Stenotrophomonas acidaminiphila*（99.93%） | | *Stenotrophomonas* |
| HD3 | *Bacillus paramycoides strain AzoM2*（100%） | | *Bacillus* |
| HD4 | *Alcaligenes faecalis*（98.65%） | | *Alcaligenes* |
| HD5 | *Gordonia rubripertincta strain N4*（100%） | | *Gordonia* |

**Figure S1. Degradation effect of different strains on OFL**

HD1

HD2

HD3

HD4

HD5

0

10

20

30

40

50

60

70

Degradation rate（%）

Strain number

The five isolated and purified OFL degrading bacteria were inoculated into the inorganic salt medium containing OFL (the concentration of OFL was 1 mg·L^-1^, 35 ℃, 120 rpm, 7 days), and the degradation effect of the five degrading bacteria on OFL within 7 days was tested. The degradation effects of HD1, HD2, hd3, HD4 and HD5 on OFL were 66.2%, 28.4%, 61.9%, 45.6% and 58.4% respectively. As shown in the figure S1, HD1 has the strongest degradation ability. After 7 days of culture, the concentration of OFL in the culture medium decreased from 1 mg·L^-1^ to 0.338 mg·L^-1^. Therefore, HD1 was selected as a high-efficiency degrading bacterium for subsequent experiments.

**3. Determination of Degradation Intermediates of OFL**

It can be seen from Figure S2 that the molecular ion peak with high response intensity can be clearly seen, and the characteristic peak of substance (a) ion in the first-order mass spectrum is m/z = 362.1519 [M + H] +, and its molecular weight is 361.1529. It is determined that the substance in the first-order mass spectrum is OFL, while the characteristic peaks of ion fragments in the second-order mass spectrum are m/z = 261.1033, 318.1612, 314.1299 and 332.1401. Therefore, it is inferred from the above analysis that the ion fragments are B, C, D, E and F respectively (table S2). Therefore, it can be inferred that the molecular formula and structure of degradation products are shown in table S2.

|  |  |
| --- | --- |
|  |  |
|  |  |

**Figure S2. The primary (a) and secondary mass spectra (b) of OFL**

**Table S2. The mass-to-nucleus ratio, molecular weight, ionic fragments and molecular structures of intermediates of OFL degradation**

| material | m/z | molecular weight | Chemical formula | Structure |
| --- | --- | --- | --- | --- |
| A | 362.1519 | 361 | C_18_H_21_FN_3_O_4_^+^ | 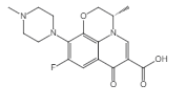 |
| B | 318.1612 | 318 | C_17_H_21_FN_3_O_2_^+^ | 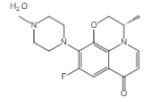 |
| C | 261.1033 | 261 | C_14_H_14_FN_2_O_2_^+^ | 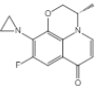 |
| D | 332.1401 | 332 | C_17_H_21_FN_3_O_3_^+^ | 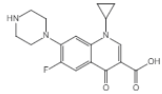 |
| E | 314.1299 | 314 | C_17_H_19_FN_3_O_2_^+^ | 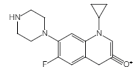 |
| F | 231.0556 | 231 | C_12_H_8_FN_2_O_2_^+^ | 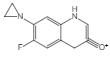 |
